# Different immunological mechanisms govern protection from experimental stroke in young and older mice with recombinant TCR ligand therapy

**DOI:** 10.3389/fncel.2014.00284

**Published:** 2014-09-25

**Authors:** Abby L. Dotson, Wenbin Zhu, Nicole Libal, Nabil J. Alkayed, Halina Offner

**Affiliations:** ^1^Neuroimmunology Research, VA Medical CenterPortland, OR, USA; ^2^Department of Neurology, Oregon Health and Science UniversityPortland, OR, USA; ^3^Department of Anesthesiology and Perioperative Medicine, Oregon Health and Science UniversityPortland, OR, USA; ^4^Knight Cardiovascular Institute, Oregon Health and Science UniversityPortland, OR, USA

**Keywords:** experimental stroke, aging, RTL1000, therapy, immune response, neuroinflammation

## Abstract

Stroke is a leading cause of death and disability in the United States. The lack of clinical success in stroke therapies can be attributed, in part, to inadequate basic research on aging rodents. The current study demonstrates that recombinant TCR ligand therapy uses different immunological mechanisms to protect young and older mice from experimental stroke. In young mice, RTL1000 therapy inhibited splenocyte efflux while reducing frequency of T cells and macrophages in the spleen. Older mice treated with RTL1000 exhibited a significant reduction in inflammatory cells in the brain and inhibition of splenic atrophy. Our data suggest age specific differences in immune response to stroke that allow unique targeting of stroke immunotherapies.

## Introduction

Stroke is a leading cause of death and disability in the United States. It is now increasingly evident that the immune response plays an important role in neurodegeneration following stroke. Splenic activation after stroke leads to an efflux of immune cells into the blood that subsequently target the brain, activate microglial cells and exacerbate the evolving brain infarct (Offner et al., 2006a,b; Seifert et al., 2012a,b). T cells are thought to be among the major contributors in post-ischemic immune response. T cells infiltrate the brain, perpetuate inflammatory conditions and contribute to increased neuronal damage following stroke (Shichita et al., 2009; Gronberg et al., 2013). Additionally, T cell knockout mice exhibit reduced infarct volume (Yilmaz et al., 2006).

Given their significant involvement in the pathogenesis of stroke, T cells are a crucial target for stroke therapy. Recombinant T-cell receptor ligand (RTL) molecules consist of the α1 and β1 domains of MHC class II molecules expressed as a single polypeptide with or without antigenic amino terminal extensions (Burrows et al., 1999; Vandenbark et al., 2003). RTLs are partial agonists that deviate autoreactive T cells to become non-pathogenic (Burrows et al., 2001; Wang et al., 2003). Previous work done by our lab has shown that RTL could treat both experimental autoimmune encephalomyelitis (EAE) and experimental stroke in mice (Burrows et al., 1998, 2001; Vandenbark et al., 2003; Huan et al., 2004; Subramanian et al., 2009; Akiyoshi et al., 2011; Dziennis et al., 2011; Benedek et al., 2014). RTL1000 is comprised of an HLA-DR2 moiety linked to human MOG-35-55 peptide (Offner et al., 2011) and has been shown to reduce infarct size when young humanized DR2 mice were treated following experimental stroke (Akiyoshi et al., 2011; Pan et al., 2014; Zhu et al., 2014).

With the potential success of RTL as a stroke therapy it becomes imperative to not only characterize the effects of RTL on the inflammatory response following stroke but to also test the RTL therapy in older mice during experimental stroke. The increased risk for stroke with age along with the substantially growing aging population will lead to an additional 3.4 million people affected by stroke by the year 2030 compared to 2012 (Ovbiagele et al., 2013). Despite the unavoidable and increasing financial burden of post-stroke care, many experimental stroke therapies have been abandoned due to their failure in the clinical setting (O'Collins et al., 2006). The inability of stroke therapy to be translated from bench to clinical success can be attributed, in part, to the lack of aging rodents used in basic research (Liu and McCullough, 2011). By examining the effects of RTL therapy on experimental stroke in both young and older mice we can more accurately determine the mechanism of therapy and predict its translation to clinical use.

## Materials and methods

### Ethics statement

The study was conducted in accordance with National Institutes of Health guidelines for the use of experimental animals, and the protocols were approved by Animal Care and Use Committee at Oregon Health & Science University and the Portland Veteran Affairs Medical Center.

### Animals

All experiments used age-matched, sexually mature (20–25 g) male HLA-DRB1^*^1502 (DR2-Tg) mice produced by Dr. Chella David (Gonzalez-Gay et al., 1996). The mice were housed and bred at the Veterans Affairs Medical Center and studies were conducted at Oregon Health and Science University.

### RTL1000 construction and production

RTL molecules consist of the α1 and β1 domains of major histocompatibility complex (MHC) II molecules and are expressed as a single polypeptide with or without antigenic amino terminal extensions (Burrows et al., 1999). RTL1000 (β1α1[5D substituted] domains of HLA-DR2 linked to human [h]MOG-35-55 peptide [MEVGWYRPPFSRVVHLYRNGK]) was constructed de novo or by sequential site-directed mutagenesis of previous constructs (Sinha et al., 2007). Protein purification was carried out as previously described (Burrows et al., 1999).

### Treatment with RTL1000

Mice were randomized to receive 0.1 mL (100 μ g) RTL1000 or 0.1 mL vehicle (5% dextrose in Tris-HCl, pH 8.5) by subcutaneous injection 4 h after the onset of reperfusion followed by doses at 24, 48, and 72 h of reperfusion for a total of four treatments each of RTL1000 or Vehicle. Both RTL1000 and vehicle treated mice were euthanized at the 96 h time-point after MCAO for further examination of tissues and cells.

### Transient middle cerebral artery occlusion

Transient focal cerebral ischemia was induced in male DR2-Tg mice for 1 h by reversible right MCAO under isoflurane anesthesia followed by 96 h of reperfusion as described previously (Zhang et al., 2008). Head and body temperature were controlled at 36.5 ± 0.5°C throughout MCAO surgery with warm water pads and a heating lamp. Occlusion and reperfusion were verified in each animal by laser Doppler flowmetry (LDF) (Model DRT4, Moor Instruments Ltd., Oxford, England). The common carotid artery was exposed and the external carotid artery was ligated and cauterized. Unilateral MCAO was accomplished by inserting a 6-0 nylon monofilament surgical suture (ETHICON, Inc., Somerville, NJ, USA) with a heat-rounded and silicone-coated (Xantopren comfort light, Heraeus, Germany) tip into the internal carotid artery via the external carotid artery stump. Adequacy of MCAO was confirmed by monitoring cortical blood flow at the onset of the occlusion with a LDF probe affixed to the skull. Animals were excluded if mean intra-ischemic LDF was greater than 30% pre-ischemic baseline. At 1 h of occlusion, the occluding filament was withdrawn to allow for reperfusion. Mice were then allowed to recover from anesthesia and survived for 96 h following initiation of reperfusion.

### Determination of infarct size

The brains were harvested after 96 h of reperfusion and sliced into four 2-mm-thick coronal sections for staining with 1.2% 2,3,5-triphenyltetrazolium chloride (TTC; Sigma, St. Louis, MO, USA) in saline as described previously (Hurn et al., 2007). The 2-mm brain sections were incubated in 1.2% TTC for 15 min at 37°C, and then fixed in 10% formalin for 24 h. Infarction volume was measured using digital imaging and image analysis software (Systat, Inc., Point Richmond, CA, USA). To control for edema, infarct volume (cortex, striatum, and hemisphere) was determined by subtraction of the ipsilateral non-infarcted regional volume from the contralateral regional volume. This value was then divided by the contralateral regional volume and multiplied by 100 to yield regional infarction volume as a percent of the contralateral region.

### Leukocyte isolation from brain and spleen

Spleens from individual MCAO-treated mice were removed and a single-cell suspension was prepared by passing the tissue through a 100 μm nylon mesh (BD Falcon, Bedford, MA). The cells were washed using RPMI 1640 and the red blood cells lysed using 1× red blood cell lysis buffer (eBioscience, Inc., San Diego, CA) and incubated for 1 min. The cells were then washed with RPMI 1640, counted on a Cellometer Auto T4 cell counter (Nexcelom, Lawrence, MA), and resuspended in staining medium [PBS containing 0.1% NaN3 and 1% bovine serum albumin (Sigma, IL)] for flow cytometry. The brain was divided into the ischemic (right) and non-ischemic (left) hemispheres, digested for 60 min with 1 mg/ml Type IV collagenase (Sigma Aldrich, St. Louis, MO) and DNase I (50 mg/ml, Roche Diagnostics, Indianapolis, IN) at 37°C with intermittent shaking. Samples were mixed with a 1 ml pipette every 15 min. The suspension was washed 1× in RPMI, resuspended in 80% Percoll overlayed with 40% Percoll, and centrifuged for 30 min at 1600 RPM. The cells were then washed twice with RPMI 1640 and resuspended in staining medium for flow cytometry.

### Analysis of cell populations by flow cytometry

All antibodies were purchased (BD Biosciences, San Jose, CA or eBioscience, Inc., San Diego, CA) as published. Four-color (FITC, PE, APC, and PerCP) fluorescence flow cytometry analyses were performed to determine the phenotypes of splenocytes and brain cells. One million cells were washed with staining medium, blocked with Anti-mouse CD16/CD32 Mouse BD Fc Block™ (BD Biosciences, San Jose) for 15 min at 4°C and then incubated with combinations of the following monoclonal antibodies: CD11b (MAC-1), CD45 (Ly-5), CD3 (145-2C11), CD11c (HL-3), CD19 (1D3), CD4 (GK1.5), CD8 (53-6.7), Ly6C (AL-21), Ly6G (RB6-8C5), CD122 (TM-β1), CD44 (IM7), CCR5 (HM-CCR5), and CD69 (H1.2F3) for 20 min at 4°C. 7-AAD was added to identify dead cells. CD4+ regulatory T cells were identified using Foxp3 (FJK-16s) and accompanying Fixation/Permeabilization reagents as per manufacturer's instructions (eBioscience, Inc., San Diego, CA). Isotype matched mAb served as a negative control. Data were collected with BD Accuri™ C6 software on a BD Accuri™ C6 (BD Biosciences, San Jose, CA).

### Cytokine determination by intracellular cytokine flow cytometry analysis

Splenocytes from individual mice were cultured at 2 × 10^6^ cells/well in a 24-well culture plate in stimulation medium (RPMI, 1% sodium pyruvate, 1% L-glutamine, 0.4% 2-β-mercaptoethanol, 2% FBS) with PMA (50 ng/ml), ionomycin (500 ng/ml), and brefeldin A (1 ul/mL) (all reagents from Sigma-Aldrich, St. Louis, MO) for 4 h. Cells were blocked, surface stained (see Section Analysis of Cell Populations by Flow Cytometry), and then fixed and permeabilized using a Cytofix/Cytoperm kit (BD Biosciences), according to manufacturer's instructions. Fixed cells were washed with 1× permeablization buffer (BD Biosciences) and stained with antibodies to IFNγ, TNFα, IL-10, IL-17, or IL-21. Isotype matched mAbs served as negative controls to establish background cytokine staining levels. Data were collected with BD Accuri™ C6 software on a BD Accuri™ C6 (BD Biosciences, San Jose, CA).

### Statistical analysis

Data are presented as mean ± s.e.m. Differences in cortical, striatal, and total (hemispheric) infarct size data was analyzed by Two-Way ANOVA, with one factor being brain region and the other factor treatment group. Spleen and brain cell counts and percentages of cellular subtypes for FACS analysis were analyzed by Student's *t*-test. Statistical significance was *p* < 0.05. Statistical analyses were performed using Prism (GraphPad, La Jolla, CA).

## Results

### RTL1000 reduces infarct volume in young and older mice

**Figure 1:**
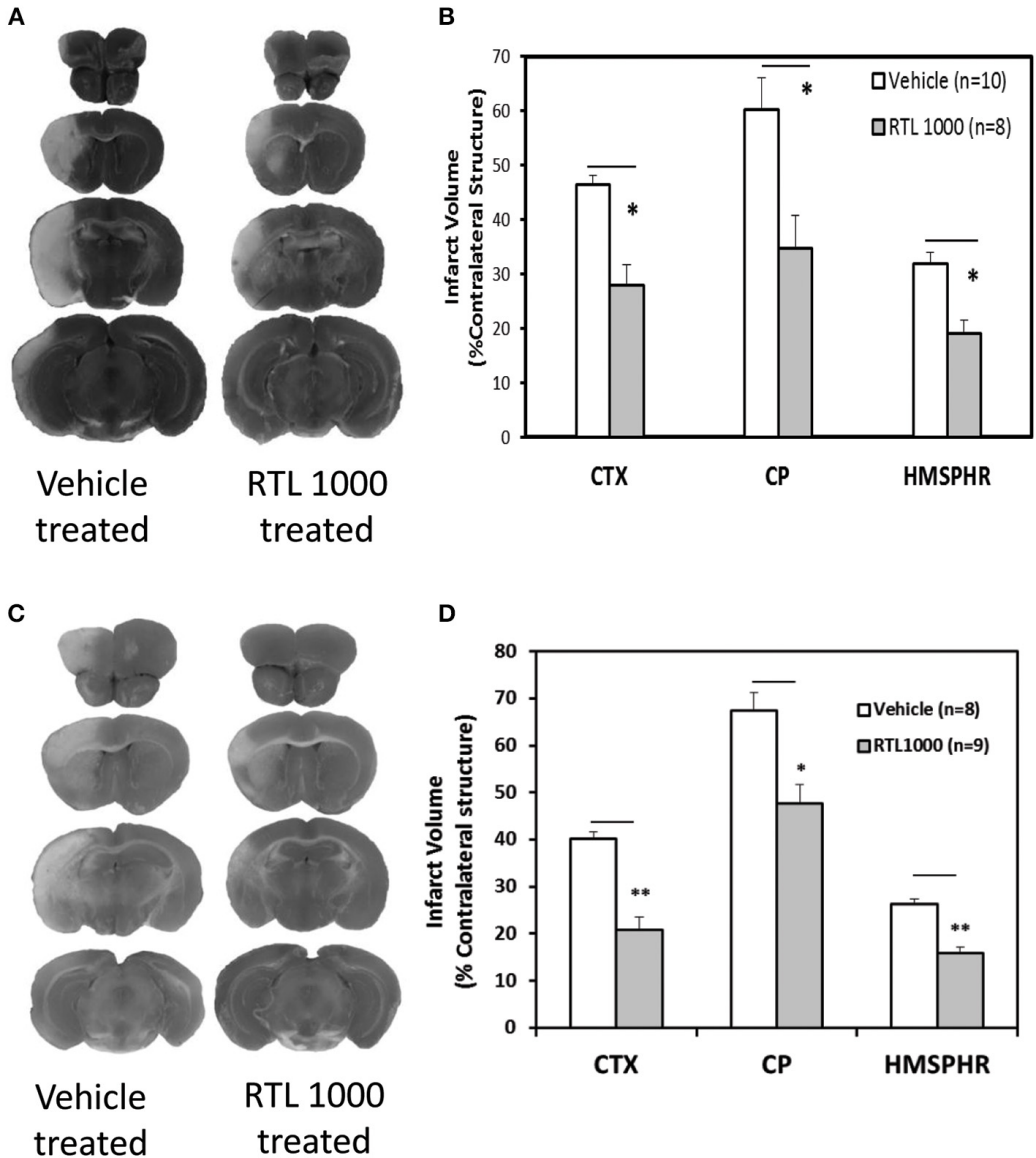
**RTL1000 reduces infarct volume in young and older mice**. Transient MCAO (60 min) male mice were treated with 100 μl vehicle or 100 μg RTL1000 given S.C. at 4, 24, 48, and 72 h after MCAO. Brains were harvested 96 h after MCAO and brain slices were stained with 2,3,5-triphenyltetrazolium chloride (TTC) **(A,C)**. Infarct volumes were measured as percentage of contralateral structure in young **(B)** and older mice **(D)**. ^*^ Indicates *p* < 0.05 and ^**^ indicates *p* < 0.01 compared with vehicle group by Two-Way ANOVA.

Eight week HLA-DR2 mice exhibited a significant reduction in infarct volume with RTL compared to vehicle (Figures 1A,B). Representative TTC-stained cerebral sections illustrate reduction in infarct in mice that received RTL after MCAO compared to vehicle in young mice (Figure 1A). Cortical infarct volume was reduced from 46.4 ±1.7% to 28 ± 3.7% and striatal infarct volume decreased from 60.1 ± 5.9% to 34.7 ± 6.1% when mice were given RTL following MCAO (Figure 1B). Total hemispheric infarct volume was also significantly reduced from 31.8 ± 2.1% to 19.1 ± 2.4% with RTL (Figure 1B). Similarly, infarct volume was significantly decreased in 16-month-old mice given RTL compared to vehicle (Figures 1C,D). Representative TTC-stained cerebral sections illustrate reduction in infarct in older mice that received RTL after MCAO compared to vehicle (Figure 1C). Cortical and striatal infarct volume went from 40.1 ±1.6% to 20.7 ± 2.8% and 67.5 ± 3.9% to 47.7 ± 4.0% respectively, when RTL was administered after MCAO (Figure 1D). Total hemispheric infarct was also significantly reduced from 26.2 ±1.2% to 16.0 ±1.3% with RTL (Figure 1D). The mortality rate of MCAO in both treatment groups was less in young mice compared to older mice while RTL reduced mortality in older mice only (Table 1). Together, these data reveal that RTL1000 improves stroke outcome in both young and older mice.

**Table 1:** **Mortality and success rate of MCAO**.

	**Animal numbers underwent MCAO**	**Number of animals died after MCAO**	**Number of animals excluded for SAH**	**Number of animals excluded for other reasons**	**Final animal numbers**
Vehicle (8 week)	13	3 (23%)	0	0	10
RTL1000 (8 week)	12	3 (25%)	0	1 (Massive cerebral hemorrhage)	8
Vehicle (16 month)	18	8 (44.4%)	2	0	8
RTL1000 (16 month)	15	5 (33.3%)	0	1 (LDF > 30%)	9

### RTL1000 reduces activated and infiltrating cells in the ischemic hemisphere of 16 month but not 8-week-old mice

We wanted to decipher the mechanism of protection with RTL1000 in young and older mice following MCAO by examining brain infiltrating immune and inflammatory cells. Cells were isolated from the ischemic hemisphere 96 h after MCAO, phenotyped and counted (Table 2). The percent of activated microglia and/or infiltrating monocytes/macrophages (CD11b+CD45^hi^) in the ischemic hemisphere after MCAO was reduced in both 8-week- and 16-month-old mice treated with RTL (Figure 2A). Absolute number of activated microglia/monocytes was also significantly reduced in the 16-month-old mice that received RTL and there was a trend reduction in young mice that received RTL (*p* = 0.068) (Figure 2B). Ischemic hemispheres of 16-month-old mice that received RTL after MCAO also exhibited a significant reduction in absolute CD3+ T cells and CD11c+ dendritic cells compared to vehicle (Figures 2C,D). Young mice exhibited a trend reduction in number of T cells in the ischemic hemisphere with RTL (*p* = 0.064) but did not have a significantly reduced number of dendritic cells after treatment with RTL compared to vehicle (Figures 2C,D). The number of infiltrating B cells in the ischemic hemisphere following MCAO with or without RTL did not display significance difference in either young or older mice (Figure 2E). Additionally, total infiltrating cells and cell death were not affected by RTL treatment in either age group (Figures 2F,G). Control (non-ischemic) hemispheres did not exhibit an increase in inflammatory parameters (data not shown). These data suggest that RTL1000 may reduce infarct volume following stroke using different mechanisms between young and older mice.

**Table 2:** **Ischemic (right) hemisphere**.

**Cell type**	**8-week-old mice**		**16-month-old mice**	
	**Vehicle**	**RTL1000**	**Vehicle**	**RTL1000**
Total cell number	3.4 × 10^5^ ± 7.4 × 10^4^	2.7 × 10^5^ ± 3.0 × 10^4^	6.0 × 10^5^ ± 8.6 × 10^4^^#^	5.4 × 10^5^ ± 6.3 × 10^4^^#^
7AAD+	2.4 × 10^4^ ± 3451	2.0 × 10^4^ ± 2671	3.8 × 10^4^ ± 3395^#^	2.7 × 10^4^ ± 3674
CD11b+ CD45^hi^	6.9 × 10^4^ ± 1.5 × 104	3.3 × 10^4^ ± 5135	6.4 × 10^4^ ± 8554	1.7 × 10^4^ ± 5891^**^
CD3+ T cells	2769 ± 591	1375 ± 107	3109 ± 624	1188 ± 457^*^
CD11c+ DC	4.1 × 10^4^ ± 9840	2.6 × 10^4^ ± 6420	2.6 × 10^4^ ± 5916	5073 ± 1017^*^^#^
CD19+ B cells	6149 ± 1526	3754 ± 734	7471 ± 719	5679 ± 508

* indicates significance compare to vehicle ^*^p ≤ *0.05*; ^**^p ≤ *0.01*.

# indicates significance compared to 8-week-old mice ^#^p ≤ *0.05*.

**Figure 2:**
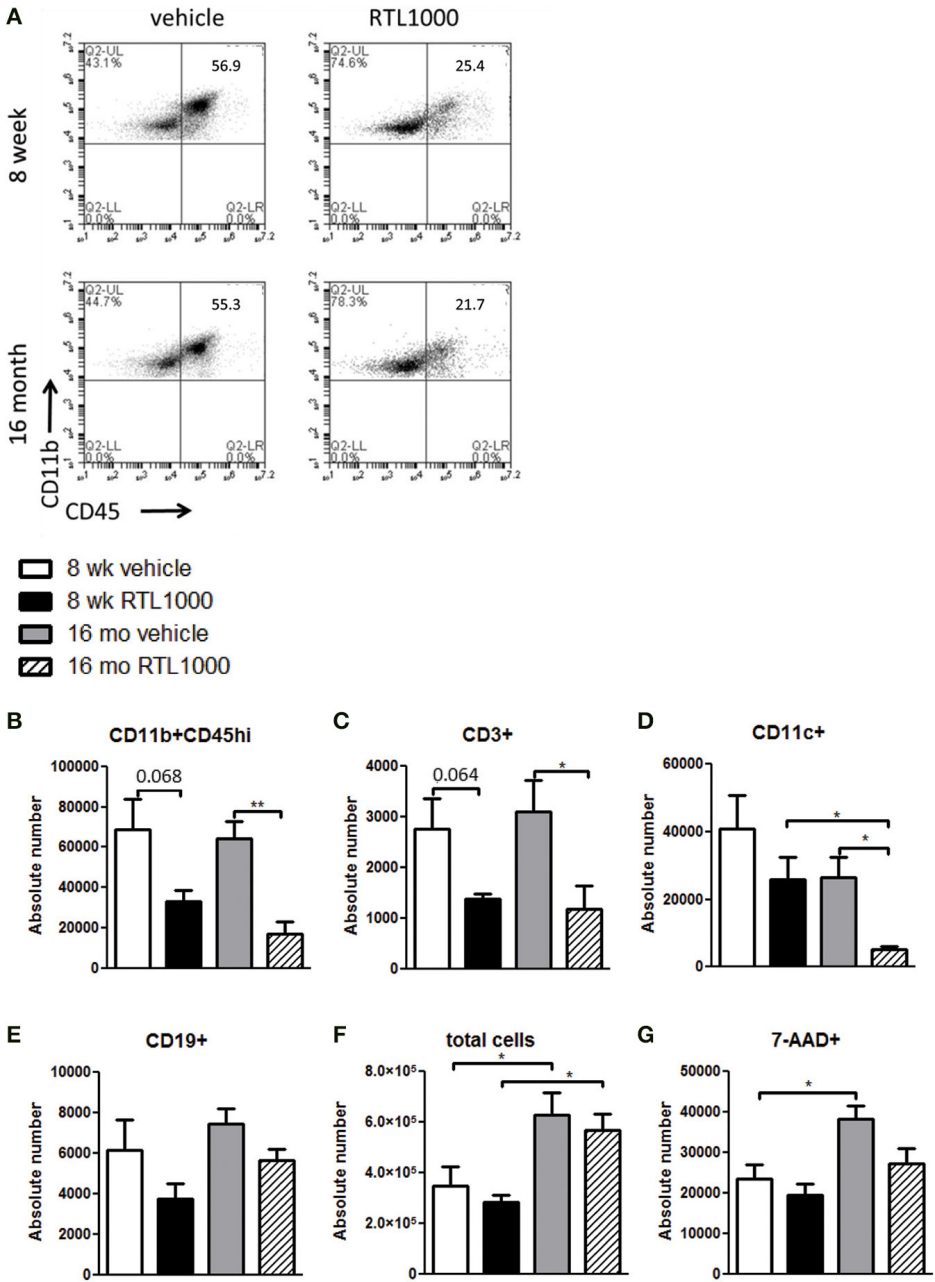
**RTL1000 reduces activated and infiltrating cells in the ischemic hemisphere of 16 month but not 8-week-old mice**. Brains from young or older male mice were harvested 96 h after MCAO. Leukocytes were isolated from the ischemic hemisphere and phenotyped by flow cytometry. Representative flow cytometry dot plots of activated microglia/monocytes **(A)**. Absolute number of each cell phenotype was calculated using phenotype percent and total cell numbers. CD11b+CD45^hi^ activated microglia/monocytes **(B)**, CD3+ T cells **(C)**, CD11c+ dendritic cells **(D)** and CD19+ B cells **(E)** in the ischemic hemisphere were determined. Total cell number was calculated by multiplying cells per microliter run through the flow cytometer by the total sample volume **(F)**. Cells were analyzed for death by 7-AAD **(G)**. Values represent mean numbers (±s.e.m.) of indicated cell subsets from 5 to 6 young mice per treatment group, from 2 to 3 separate experiments and 4 older mice per group, from 2 separate experiments each. ^*^ Indicates *p* < 0.05 and ^**^ indicates *p* < 0.01 compared with vehicle group by *t*-test.

### Splenic atrophy is prevented with RTL1000

**Figure 3:**
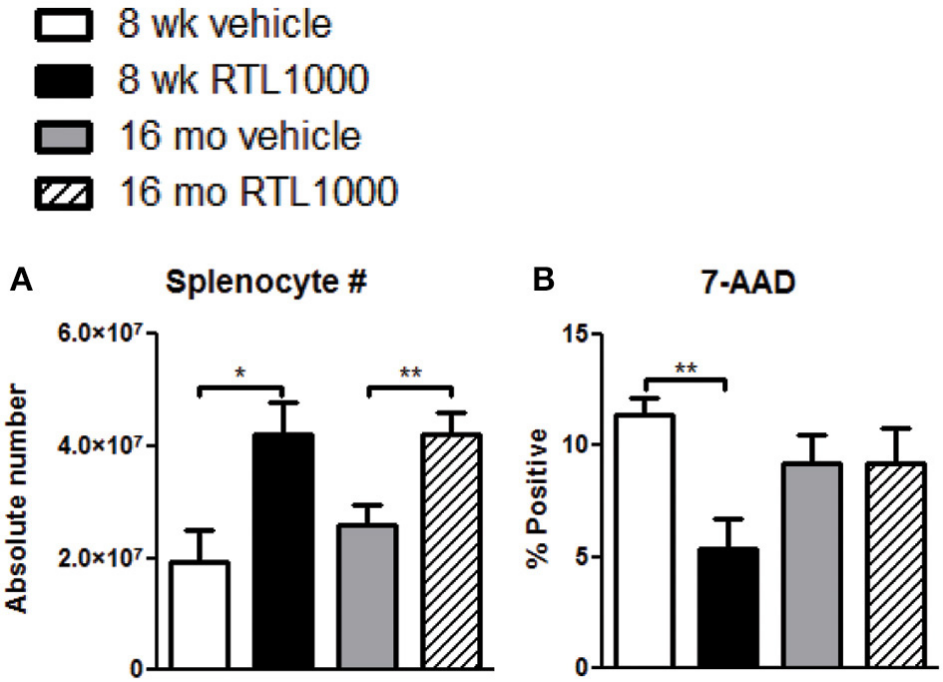
**Splenic atrophy is prevented with RTL1000**. Spleens were harvested 96 h after MCAO. Splenocytes were isolated and counted by a Cellometer T4 and total splenocyte number was calculated **(A)**. Values represent mean numbers (±s.e.m.) of 6 young vehicle, 5 young RTL, 12 older vehicle, and 13 older RTL mice. Splenocyte death was determined by 7-AAD fluorescence **(B)**. Values represent mean numbers (±s.e.m.) of 5 young mice from each treatment group, 6 older vehicle and 9 older RTL mice. ^*^ Indicates *p* < 0.05 and ^**^ indicates *p* < 0.01 compared with vehicle group by *t*-test.

To determine other possible mechanisms of RTL1000 protection following stroke, we examined immune parameters in the periphery. The spleen is known to have a major impact on stroke. Our lab and others have established that splenic atrophy occurs following stroke, releasing splenocytes into systemic circulation and exacerbating neurodegeneration (Offner et al., 2006b; Seifert et al., 2012a). Spleens were harvested 96 h after MCAO and splenocytes were counted and evaluated for viability (Table 3). RTL treatment significantly inhibited splenic atrophy in both 8-week- and 16-month-old mice (Figure 3A). Splenocyte number correlated inversely with cell death in young mice as determined by 7-AAD staining. Young mice treated with RTL exhibited a significant decrease in splenocyte death compared to vehicle while older mice showed no change in 7-AAD positive cells between the treatments (Figure 3B).

**Table 3:** **Splenocyte number and viability**.

	**8-week-old mice**		**16-month-old mice**	
	**Vehicle**	**RTL1000**	**Vehicle**	**RTL1000**
Splenocyte number	1.9 × 10^7^ ± 5.5 × 10^6^	4.2 × 10^7^ ± 5.6 × 10^6^^*^	2.6 × 10^7^ ± 3.8 × 10^6^	4.2 × 10^7^ ± 4.1 × 10^6^^**^
Cell death %	11.3 ± 0.8	5.3 ± 1.36^**^	9.2 ± 1.28	9.2 ± 1.63

* indicates significance compare to vehicle ^*^p ≤ 0.05 ^**^p ≤ 0.01.

### RTL1000 primarily affects peripheral immune subsets in younger mice

We also characterized immune cell phenotypes within the spleens of young and old mice, with and without RTL treatment following MCAO (Table 4). We found a significant reduction in the frequency of CD4+ T cells with RTL treatment in both young and old mice (Figure 4A). RTL treatment also significantly reduced the percent of CD8+ T cells, CD3+ total T cells, and CD11b+ myeloid cells in young mice only (Figures 4B–D). Furthermore, only the Ly6C+, not Ly6G+, subset of CD11b cells were reduced with RTL in young mice indicating that the reduction of total CD11b cells was due to a reduction in monocytes/macrophages not CD11b+ neutrophils (Figure 4E, Table 4). The percent of CD19+ B cells in the spleens of young RTL treated mice following MCAO was significantly greater compared to vehicle (Figure 4F). Collectively, in young mice RTL treatment reduces the percent of T cells and monocytes/macrophages, both of which are known to contribute to neurodegeneration following stroke (Campanella et al., 2002; Yilmaz et al., 2006), while increasing potentially protective B cells (Bodhankar et al., 2014). The data also demonstrate that RTL significantly impacts multiple subsets of splenocytes in young mice while having much less impact on the splenocytes of older mice after MCAO, supporting the hypothesis that RTL1000 targets different immune pathways in younger and older mice.

**Table 4:** **Cell types in the spleen**.

	**8-week-old mice**		**16-month-old mice**	
**Cell type**	**Vehicle**	**RTL1000**	**Vehicle**	**RTL1000**
Total CD3+ T cells	34.2 ± 1.6	27.7 ± 1.8^*^	21.0 ± 2.1^##^	18.4 ± 0.7^###^
CD4+ T cells	18.7 ± 1.2	15.1 ± 0.6^*^	12.8 ± 0.8^##^	9.8 ± 0.3^**^^###^
CD8+ T cells	13.9 ± 0.9	9.9 ± 1.2^*^	10.0 ± 1.0^#^	10.0 ± 0.9
CD11b+ myeloid cells	4.9 ± 0.4	3.6 ± 0.4^*^	3.2 ± 0.2^##^	3.5 ± 0.2
CD11b+Ly6G+ neutrophils	4.9 ± 0.4	3.8 ± 0.5	4.7 ± 0.3	4.3 ± 0.3
CD11b+Ly6C+ monocytes	3.5 ± 0.3	2.2 ± 0.4^*^	2.6 ± 0.3^#^	2.9 ± 0.2
CD11c+ dendritic cells	1.6 ± 0.1	1.8 ± 0.2	1.3 ± 0.1	1.9 ± 0.2
CD19+ B cells	42.8 ± 2.7	53.7 ± 2.0^*^	59.0 ± 1.5^##^	62.3 ± 1.5^##^

* indicates significance compare to vehicle ^*^p ≤ 0.05; ^**^p ≤ 0.01.

# indicates significance compared to 8-week-old mice ^#^p ≤ 0.05; ^##^p ≤ 0.01; ^###^p ≤ 0.001.

**Figure 4:**
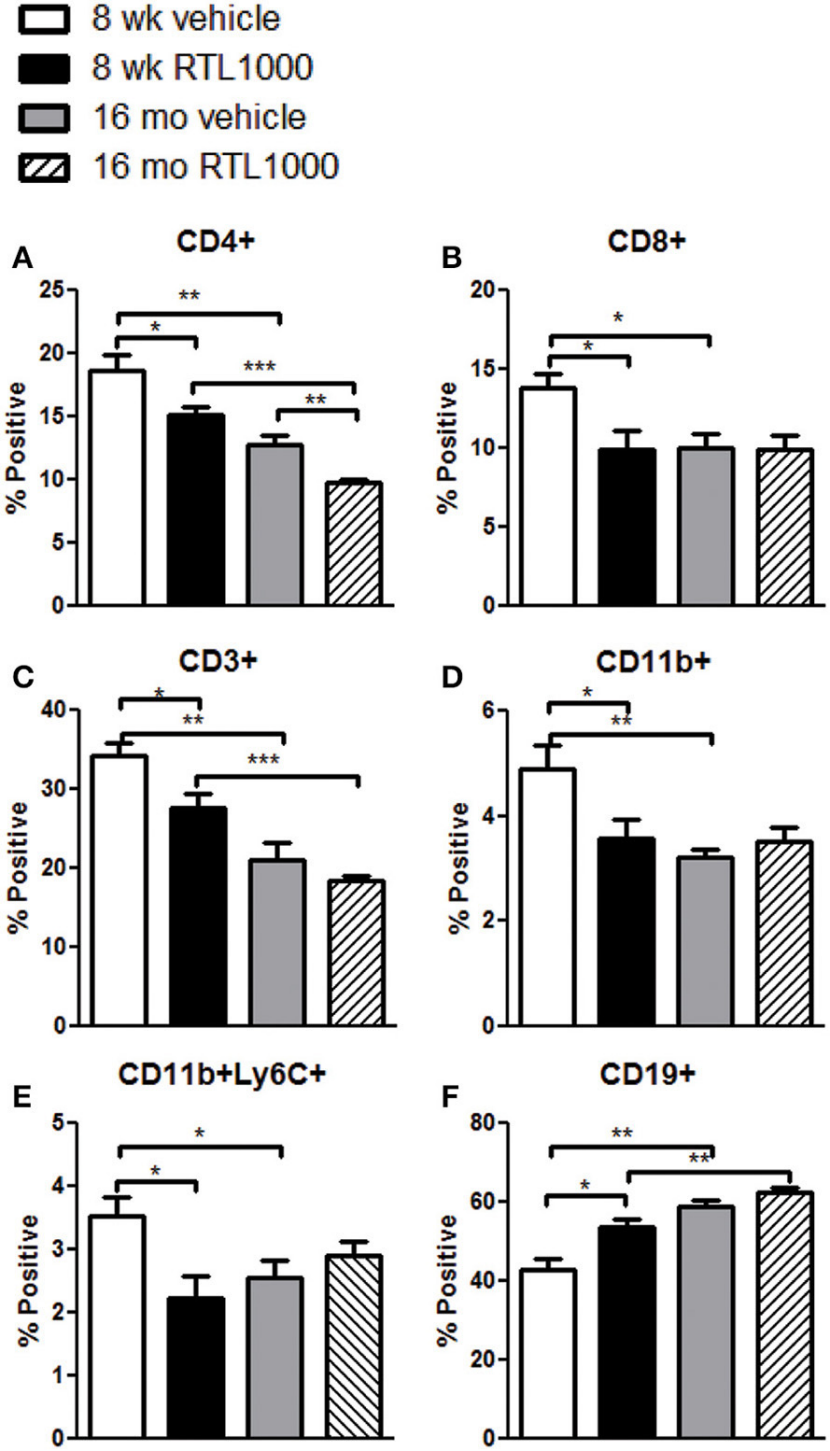
**The effect of RTL1000 on peripheral immune subsets in younger vs. older mice**. Spleens were harvested 96 h after MCAO and immunophenotyped by flow cytometry. The frequency of CD4+ T cells **(A)**, CD8+ T cells **(B)**, CD3+ T cells **(C)**, CD11b+ monocytes/neutrophils **(D)**, CD11b+Ly6C+ monocytes/macrophages **(E)**, and CD19+ B cells **(F)** were determined. Values represent mean numbers (±s.e.m.) of 5 young mice from each treatment group, 4–6 older vehicle and 9 older RTL mice. ^*^ Indicates *p* < 0.05, ^**^ indicates *p* < 0.01, and ^***^ indicates *p* < 0.001 compared with vehicle group by *t*-test.

### Peripheral immune properties change with age following stroke

In addition to the differential effects of RTL on immune subsets after MCAO between the age groups, we also observed baseline immunological discrepancies in the periphery of young and older mice. The frequency of CD3+ total T cells and CD4+ helper T cells were significantly greater in 8-week-old vehicle and RTL treated mice following MCAO than in 16-month-old vehicle and RTL treated mice, respectively (Figures 4A,C). CD8+ T cells were significantly greater in younger MCAO vehicle mice compared older MCAO vehicle mice (Figure 4B). Total CD11b cells and CD11b+Ly6C+ monocyte/macrophage subset were both significantly higher in the spleens of young mice that received vehicle after MCAO vs. their older counterparts (Figures 4D,E). Additionally, the frequency of CD19+ B cells was significantly less in the spleen following MCAO in both vehicle and RTL treated younger mice than older mice. Innate differences in the peripheral immune composition between 8-week- and 16-month-old mice following MCAO may give insight into the different possible targets of RTL1000 following stroke.

### Splenic CD4+ regulatory T cells increase with age in MCAO mice while CD8+ regulatory T cells are increased by RTL1000 treatment

CD4+ regulatory T cells are upregulated following stroke (Offner et al., 2006b) and CD8+ regulatory T cells are known to suppress CD4+ and CD8+ T cell proliferation and IFNγ production by secreting IL-10 (Rifa'i et al., 2008). Therefore, we wanted to characterize regulatory populations in the spleen to determine their role in the periphery following stroke (Table 5). We saw no difference in CD4+ regulatory T cells (CD4+Foxp3+) in young or older mice treated with or without RTL after stroke (Figure 5A). However, there was a significant increase in regulatory CD4+ T cells following MCAO in older compared to younger RTL treated mice and the same trend (*p* = 0.0675) is seen with the vehicle mice (Figure 5A). Regulatory CD8+ T cells (CD122+IL-10+) were significantly increased in the spleens of young mice treated with RTL after MCAO but not in older mice (Figure 5B).

**Table 5:** **Regulatory cells in the spleen**.

**Cell type**	**8-week-old mice**		**16-month-old mice**	
	**Vehicle**	**RTL1000**	**Vehicle**	**RTL1000**
CD4+Foxp3+	15.42 ± 2.0	16.12 ± 0.8	21.63 ± 2.0	20.2 ± 1.4^#^
CD8+CD122+ IL-10+	3.9 ± 0.4	5.2 ± 0.4^*^	4.2 ± 0.2	3.4 ± 0.8

* indicates significance compare to vehicle ^*^p ≤ 0.05.

# indicates significance compared to 8-week-old mice ^#^p ≤ 0.05.

**Figure 5:**
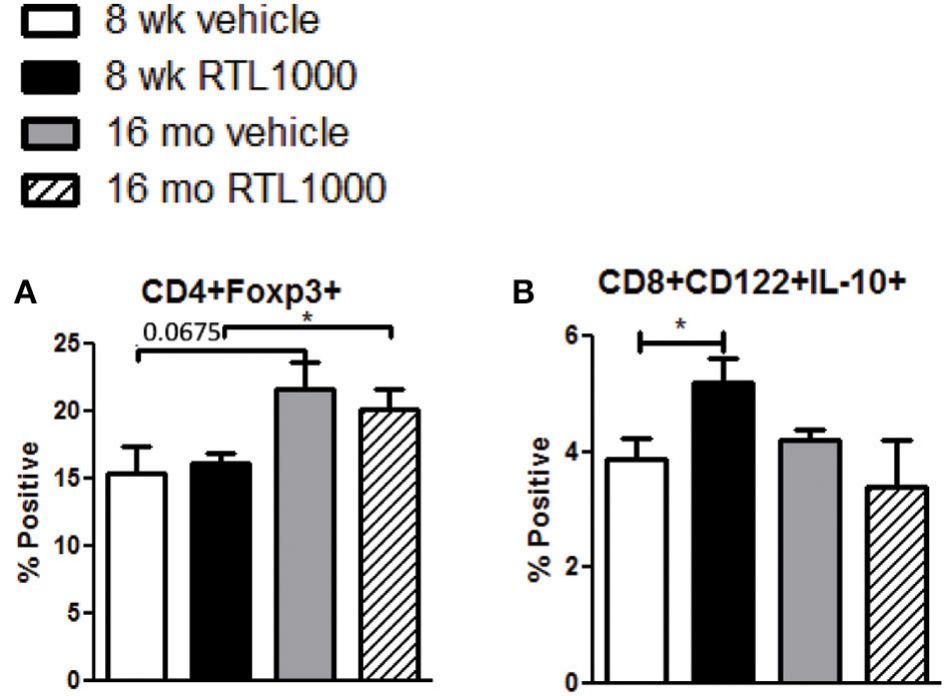
**Splenic CD4+ regulatory T cells increase with age in MCAO mice while CD8+ regulatory T cells are increased by RTL1000 treatment**. Spleens were harvested 96 h after MCAO and immunophenotyped by flow cytometry. CD4+ regulatory T cells were gated on CD4+ positive splenocytes and determined by Foxp3 expression compared to isotype **(A)**. Values represent mean numbers (±s.e.m.) of 5 young mice from each treatment group, 4 older vehicle and 7 older RTL mice. CD8+ regulatory T cells were gated on the CD8+CD122+ subset and determined by IL-10 expression compared to isotype **(B)**. Values represent mean numbers (±s.e.m.) of 5 young mice from each treatment group, 3 older vehicle and 5 older RTL mice. ^*^ indicates *p* < 0.05.

### T cells in the spleen of younger mice increase activation marker CD69 while older mice display elevated CD44 expression following MCAO

**Figure 6:**
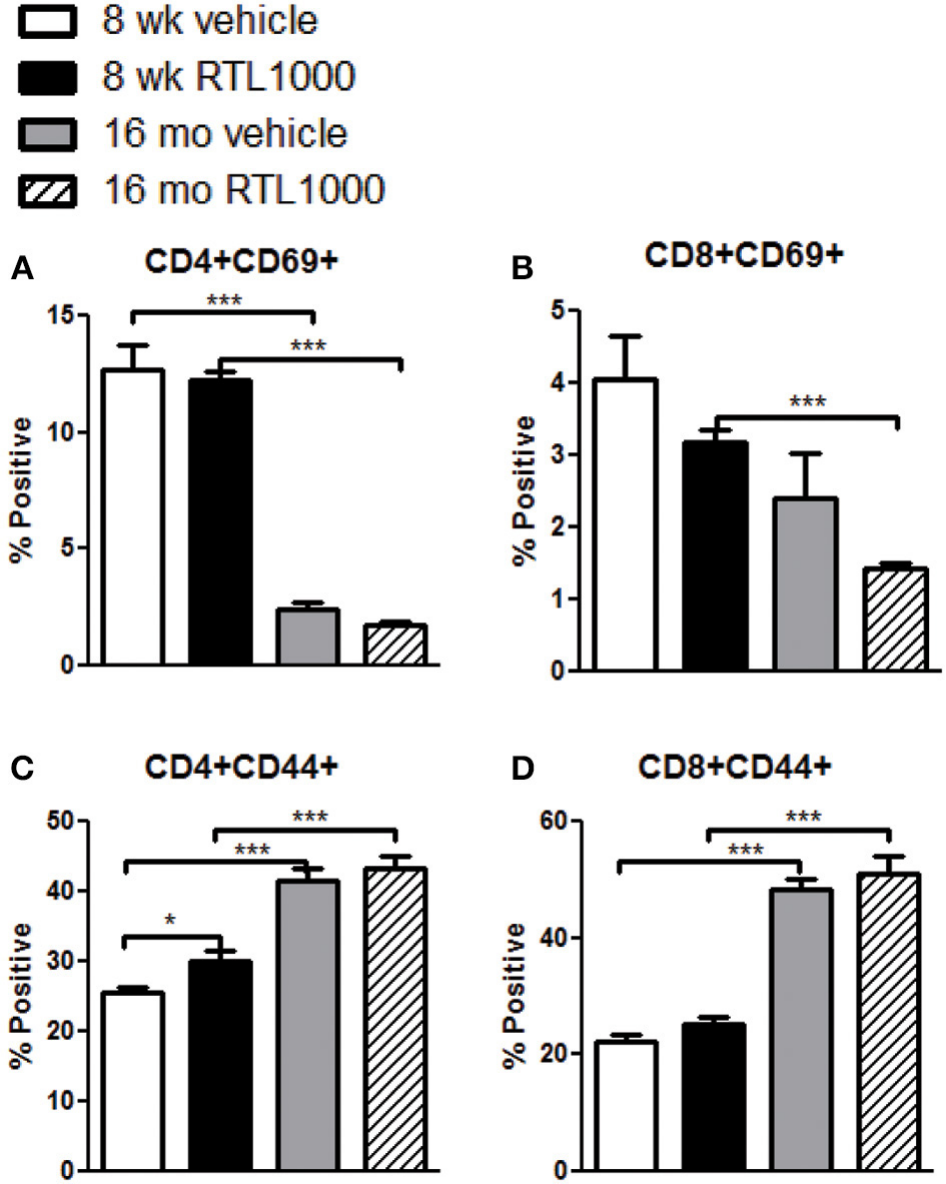
**T cells in the spleen of younger mice increase activation marker CD69 while older mice display elevated CD44 expression following MCAO**. Spleens were harvested 96 h after MCAO. Early T cell activation was determined by gating on CD4+ **(A)** or CD8+ **(B)** subsets and measuring expression of early activation marker, CD69. Effector/central memory was determined by gating on CD4+ **(C)** or CD8+ **(D)** subsets and measuring expression of CD44. Values represent mean numbers (±s.e.m.) of 5 young mice from each treatment group, 7 older vehicle and 9 older RTL mice. ^*^ indicates *p* < 0.05 and ^***^ indicates *p* < 0.001 compared with vehicle group by *t*-test.

In addition to identifying the frequency of different immune subsets in the spleen, functionality of those subsets following stroke and RTL1000 treatment was also examined. CD69 is the earliest activation-inducible cell surface molecule and is involved in cell signaling and lymphocyte proliferation (Serra et al., 1996). CD44 is an adhesion receptor on T cells that is upregulated following initial activation and remains highly expressed on effector and memory T cells (Baaten et al., 2012). No significant differences were seen in CD69 or CD44 expression on T cells between vehicle and RTL treatment in either mouse group with the exception of an increase of CD44 with RTL treatment on CD4+ T cells T cells in young mice (Figures 6A–D). There was, however, a significant or trend increase in CD69 expression on CD4 and CD8 T cells in young mice compared to older mice (Figures 6A,B). Furthermore, CD44 was significantly increased on splenic CD4 and CD8 T cells from older mice after MCAO compared to younger mice (Figures 6C,D). We hypothesize that T cells in the younger mice are able to become activated efficiently, thus the increased CD69 expression after MCAO, yet elevated frequencies of effector/memory T cells observed in older mice lead to a sustained greater number of T cells with high CD44 expression. An examination of migration marker and chemokine receptor CCR5 indicated no change in expression after MCAO between vehicle and RTL treatment or age groups (Table 6).

**Table 6:** **Activation/migration markers on T cells**.

**Cell type/Marker**	**8-week-old mice**		**16-month-old mice**	
	**Vehicle**	**RTL1000**	**Vehicle**	**RTL1000**
CD4+CD69+	12.7 ± 1.1	12.3 ± 0.4	2.4 ± 0.4^###^	1.8 ± 0.1^###^
CD8+CD69+	4.1 ± 0.6	3.2 ± 0.2	2.4 ± 0.6	1.4 ± 0.1^###^
CD4+CD44+	25.5 ± 0.8	30.1 ± 1.5^*^	41.5 ± 1.7^###^	43.3 ± 1.9^###^
CD8+CD44+	22.3 ± 1.3	25.1 ± 1.3	48.5 ± 1.7^###^	51.0 ± 3.0^###^
CD3+CCR5+	8.5 ± 1.3	9.3 ± 0.4	8.6 ± 1.3	8.4 ± 0.4

* indicates significance compare to vehicle ^*^p ≤ 0.05.

# indicates significance compared to 8-week-old mice ^###^p ≤ 0.001.

### Inflammatory cytokines are elevated in spleens from older mice after MCAO

Inflammatory cytokines play an integral role in the effector functions of the immune response. Spleen derived IFNγ directly contributes to neurodegeneration related to stroke (Yilmaz et al., 2006; Seifert et al., 2012b). TNFα, IL-17, and IL-21 have also have also been linked to stroke progression (Barone et al., 1997; Li et al., 2001; Pan and Kastin, 2007; Gelderblom et al., 2012; Swardfager et al., 2013; Clarkson et al., 2014). We found no significance difference in the frequency of specific immune cells secreting IFNγ, TNFα, IL-17, or IL-21 between vehicle and RTL treated mice with either young or old MCAO mice (Table 7). IL-21 production by splenic CD4+ T cells was significantly greater in older mice compared to younger mice following MCAO for the RTL treated group only and there was no difference in IL-17 production between the two age groups with either vehicle or treatment (Table 7). IFNγ producing CD4+ and CD8+ T cells were significantly greater in the spleens of older mice than younger mice following MCAO (Figures 7A,C,D). Similarly, TNFα positive CD4+ and CD11b+ cells were also increased in the spleens of older mice than younger mice (Figures 7B,E,F). We believe that 96 h post-MCAO may be a window of time where memory and effector immune cells which are quick to reactivate have accumulated in older mice, thus leading to immediate and amplified cytokine production compared to the higher frequency of naïve cells in young mice.

**Table 7:** **Cytokine production**.

**Cell type/Cytokine**	**8-week-old mice**		**16-month-old mice**	
	**Vehicle**	**RTL1000**	**Vehicle**	**RTL1000**
CD4+IFNγ+	4.8 ± 0.7	5.6 ± 0.3	10.2 ± 1.7^#^	9.5 ± 1.6^#^
CD8+IFNγ+	6.0 ± 1.2	7.0 ± 0.4	21.7 ± 1.5^###^	22.1 ± 5.1^#^
CD4+TNFα+	7.2 ± 2.1	6.9 ± 1.4	30.7 ± 1.9^###^	33.1 ± 3.2^###^
CD11b+TNFα+	7.0 ± 0.6	7.1 ± 0.5	33.1 ± 2.6^###^	39.4 ± 1.4^###^
CD4+IL-17+	1.3 ± 0.1	1.8 ± 0.2	1.6 ± 0.6	1.7 ± 0.4
CD4+IL-21+	1.8 ± 0.3	1.6 ± 0.2	3.5 ± 1.2	3.0 ± 0.5^#^

# indicates significance compared to 8-week-old mice ^#^p ≤ 0.05; ^###^p ≤ 0.001.

**Figure 7:**
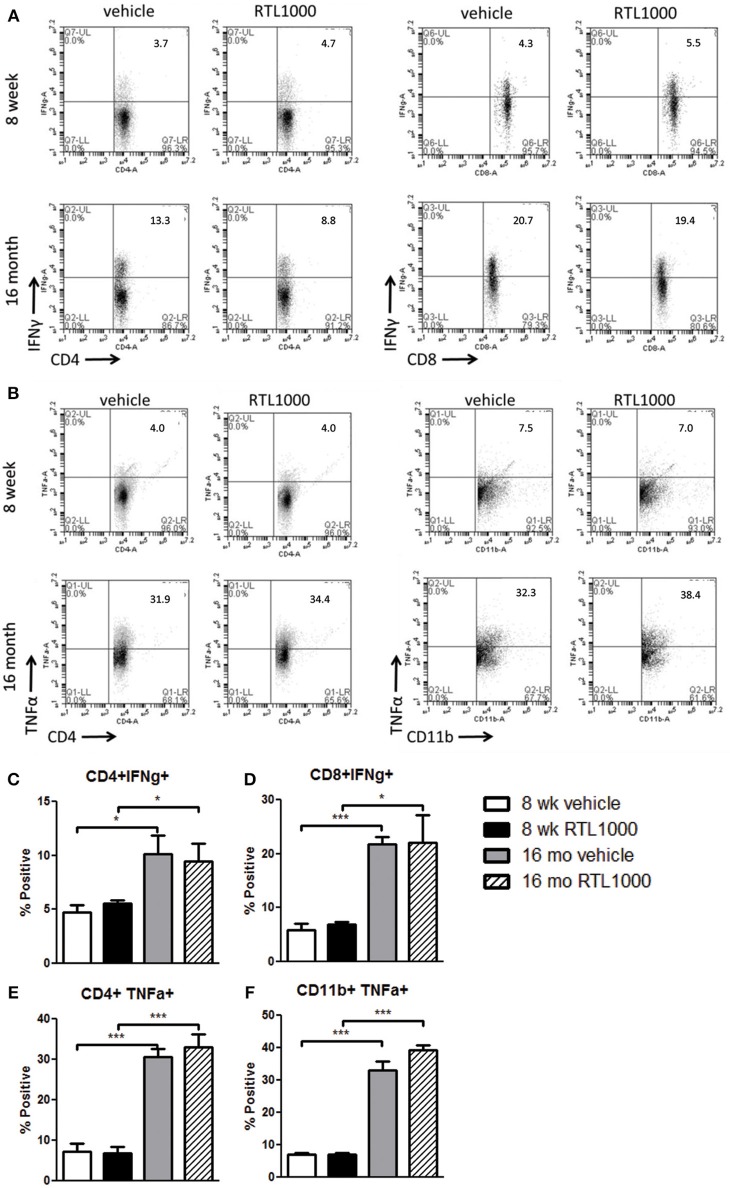
**Inflammatory cytokines are elevated in spleens from older mice after MCAO**. Spleens were harvested 96 h after MCAO. Representative flow cytometry dot plots of IFNγ **(A)** and TNFα **(B)** producing immune subsets. IFNγ production was determined by gating on CD4+ **(C)** or CD8+ **(D)** subsets and measuring IFNγ positive cells compared to isotype. TNFα production was determined by gating on CD4+ **(E)** or CD11b+ **(F)** subsets and measuring TNFα positive cells compared to isotype. Values represent mean numbers (±s.e.m.) of 5 young mice from each treatment group, 3 older vehicle and 5 older RTL mice. ^*^ Indicates *p* < 0.05 and ^***^ indicates *p* < 0.001 compared with vehicle group by *t*-test.

## Discussion

In the next few decades nearly 4% of the population will be affected by stroke (Ovbiagele et al., 2013) leading to increase in stroke-related mortality and disability and an even greater need for stroke therapies. Furthermore, understanding how the body responds to stroke as we age is equally as imperative. Previous work done by our lab and others has outlined the contributing immunological factors that influence neurodegeneration following stroke. In young male mice, MCAO induces peripheral immune pro-inflammatory activation that triggers immune cell efflux from the spleen into circulation, some of which go on to target the brain (Offner et al., 2006a,b; Seifert et al., 2012a). Despite the over 1000 neuroprotectants that have been successful in preclinical studies and more than 100 clinical trials initiated, there has been an astounding failure in translation of those neuroprotectants to successful stroke therapies in humans (Turner et al., 2013). One contributing factor to the lack of success in clinical stroke therapy is the underrepresentation of older mice in therapy based research to more accurately represent the age of most humans afflicted by stroke.

RTL therapy has been shown by our lab to be a promising neuroprotectant therapy for stroke in young male mice (Akiyoshi et al., 2011; Dziennis et al., 2011). Here we report that RTL1000 similarly reduced infarct volume in older and younger HLA-DR2 male mice when administered after the onset of MCAO. RTL1000 also reduced the mortality rate of MCAO in older mice. Cerebral ischemia induces activation of resident microglia and an influx of peripheral leukocytes into the brain (Huang et al., 2006). In older mice, RTL treatment significantly reduced the absolute number of activated monocytes/microglia, T cells, and dendritic cells in the ischemic hemisphere following MCAO. Although these cell numbers were also reduced with RTL in young mice after MCAO, the differences remained insignificant. RTL did not affect ischemic total infiltrating cell number or cell death, yet both were greater in older mice compared to younger mice after MCAO indicating possible differences in how young and older individuals respond to stroke.

The peripheral inflammatory response to stroke has been extensively studied and can give crucial insight into the mechanisms of neuroprotection of stroke therapy. One of the hallmarks of the immune response to stroke is splenic atrophy (Offner et al., 2006b). RTL1000 therapy restored total splenocyte numbers in young and older mice after MCAO compared to vehicle indicating a therapy based prevention of splenic atrophy in both age groups. There is an inverse correlation of percent cell death and total cell number in the spleen of young mice. The same trend of increased splenocyte death following MCAO was also observed in young male C57BL/6 mice by our lab (Offner et al., 2006b). RTL therapy did not, however, reduce splenocyte death in older mice compared to vehicle. These data suggest that RTL therapy prevents splenic atrophy by preventing cell death and likely by inhibiting splenocyte migration in young mice while only preventing splenocyte migration in older mice.

Previous work published by our lab revealed that in addition to a total reduction of cell numbers in the spleen, MCAO also contributes to a shift in the frequencies of the different immune subsets that comprise the spleen (Offner et al., 2006b). Specifically, we saw a significant increase in CD4+, CD8+, and CD3+ T cells and a significant decrease of B cells in the spleens of C57BL/6 mice 96 h after MCAO compared to sham (Offner et al., 2006b). RTL treatment significantly decreased the frequency of CD4+, CD8+, and CD3+ T cells and increased B cells in the spleen of young mice following MCAO suggesting that in young mice, RTL treatment counteracts changes in immune cell subsets that occur in the spleen after stroke. In addition to stroke induced changes in T and B cell frequencies in the spleen, RTL treatment led to a modest but significant reduction in monocytes/macrophages in young mice. Circulating monocytes and macrophages are recruited to the ischemic brain 3–7 days after stroke where they contribute to neurodegeneration and recruitment of additional pathogenic immune cells (Chiba and Umegaki, 2013). Since we do not observe a recruitment of activated monocytes/macrophages into the ischemic brain of RTL treated young mice after MCAO, we can hypothesize that the reduction of monocytes/macrophages in the spleen is due to inhibition of activation-induced cell expansion and not cellular migration out of the spleen.

The changes in splenocyte subsets between RTL treated and vehicle control mice were not the only remarkable differences observed in the spleen 96 h after MCAO. In fact, of the six immune subsets that were significantly affected by RTL treatment after MCAO in young mice, CD4+ T cells were the only cell group that were also significantly reduced with older, RTL treated MCAO mice compared to vehicle. Additionally, major differences were observed in peripheral immune cell frequencies after stroke in young vs. older control mice. CD4+, CD8+, and CD3+ T cells, total CD11b+ and CD11b+Ly6C+ monocytes/macrophages were all significantly reduced in the older control MCAO mice compared to the young control mice. RTL treated groups followed the same pattern for CD4+ and CD3+ T cells. CD19+ B cells were significantly elevated in both the control and RTL groups of older mice following MCAO compared to young mice. Such notable disparities in peripheral immune frequencies continue to confirm that younger and older mice respond to stroke and RTL treatment following stroke through different immunological mechanisms.

The frequency of CD4+ Foxp3+ regulatory cells in the spleen increases after MCAO (Offner et al., 2006b) although there are contradicting data on whether CD4+ regulatory T cells diminish or exacerbate stroke related neuronal damage (Schabitz, 2013; Xu et al., 2013). Recent studies have reported that CD4+ Tregs play a protective role against damage following stroke (Liesz et al., 2009; Planas and Chamorro, 2009; Li et al., 2013), while others demonstrate Tregs as harmful promoters of neurodegeneration (Kleinschnitz et al., 2013a; Kleinschnitz and Wiendl, 2013b) or find that Tregs do not influence stroke (Ren et al., 2011; Gu et al., 2012; Stubbe et al., 2013). The conflicting reports of CD4+ Tregs and stroke thus far indicate that there is still much unknown about the regulation of the immune response after stroke and even less known about Treg response to stroke in older subjects. In our study RTL did not affect the frequency of CD4+Foxp3+ Tregs in either older or younger mice. There was, however, a trending increase (*p* = 0.0675) in Tregs in older vehicle vs. younger vehicle mice and a significant increase in RTL treated mice compared to younger RTL mice. The increase in spleen derived Tregs after MCAO in older mice agrees with multiple reports of Treg increase with age contributing to immune senescence (Sharma et al., 2006; Rosenkranz et al., 2007; Lages et al., 2008; Williams-Bey et al., 2011; Raynor et al., 2012). There are far fewer reports on the effect of regulatory CD8+ T cells during and after stroke. CD8+ Tregs co-express CD122 and kill/suppress effector cells via perforin and immunosuppressive cytokines, such as IL-10 (Wang and Alexander, 2009). IL-10 producing CD8+CD122+ Tregs have been directly correlated with a decrease in infarct after MCAO (Banerjee et al., 2013; Bodhankar et al., 2013). CD8+CD122+IL-10+ cells were significantly greater in the periphery of young RTL treated mice but not older RTL treated mice compared to their vehicle counterparts. Although infarct sizes were significantly less with RTL treatment of both age groups, only older mice had a significant reduction in activated microglia/monocytes, T cells, and dendritic cells compared to vehicle. We speculate that CD8+ regulatory cells from the periphery of RTL treated older mice had already migrated to the brain, thus reducing activated immune cells in the ischemic hemisphere, while remaining in the spleen of younger RTL treated mice.

CD69 is the earliest inducible cell surface antigen expressed with T cell activation. Although we observed no change in CD69 expression in mice that received RTL treatment after MCAO, there were significantly less CD69+ T cells from older mice after MCAO. In humans, aged subjects have a lower proportion of recently activated CD69+ T cells compared to younger controls and fail to upregulate CD69 expression following stimulation through CD3, PHA or PMA/Ionomycin as effectively as younger controls (Serra et al., 1996; Schindowski et al., 2002). Therefore, the discrepancy in CD69 expression between older and younger mice can be attributed to age related immune senescence in T cell activation. CD44 is a widely expressed adhesion receptor that becomes upregulated and maintained on T cells after antigen specific activation. CD44 is commonly associated with the effector memory and central memory T cell populations and has numerous functions. We were surprised to see an elevated level of CD44 on CD4+ T cells from younger mice that had received RTL treatment after MCAO. Although commonly known for its role in promoting the immune response, cell migration and T cell proliferation, CD44 is also involved in various regulatory mechanisms such as maintaining functional Tregs and cell survival (Baaten et al., 2010). CD44 has also been shown to be involved in limiting and resolving inflammation (Johnson and Ruffell, 2009). We speculate the increase in CD44 on CD4+ T cells from younger mice that received RTL after MCAO could be linked to a regulatory role that assisted in the decrease of neuroinflammation and splenic atrophy. The percent of CD44+ and memory T cells increases with age (Barrat et al., 1995; Naylor et al., 2005); therefore the significantly greater CD44 expressing CD4 and CD8 T cells in the spleen of older vehicle or RTL treated mice after MCAO is likely indicative of a larger effector and central memory population.

Inflammatory cytokines are well-known to play a destructive role in brain inflammation following stroke. Spleen derived IFNγ is directly linked to neurodegeneration (Seifert et al., 2012b) and TNFα promotes inflammation and leukocyte infiltration into the brain, thus increasing infarct size (Feuerstein et al., 1994; Barone et al., 1997). The neuroprotection observed with RTL therapy in both young and older mice cannot be attributed to a change in peripheral cytokine secretion as demonstrated in Figure 7. Interestingly, with both treatment and vehicle, IFNγ or TNFα production by CD4+ and CD8+ T cells or CD4+ T cells and CD11b+ macrophages, respectively, was significantly greater in older mice compared to young mice after MCAO. Both IFNγ and TNFα production increase with age in CD4+/CD8+ T cells and mononuclear cells, respectively (Fagiolo et al., 1993; Bandres et al., 2000; Yen et al., 2000). The abundant amount of memory cells in older compared to younger individuals, which we also observed, requires less secondary stimulus for activation and cytokine secretion and is hypothesized to be partially responsible for the cytokine elevation with age. Although the increase in IFNγ and TNFα in the older mice did not lead to an increase of infarct size compared with young mice, there were significantly greater total cells and cell death in the ischemic hemisphere of 16-month-old mice.

To summarize, the current study demonstrates that treatment with RTL1000 following MCAO significantly reduces infarct volume in 16-month-old mice similar to 8-week-old mice. However, the mechanism of neuroprotection is different between older and younger mice. RTL1000 significantly reduced infiltrating leukocytes in the brain of older mice while significantly reducing cell death and altering the frequency of specific splenocyte subsets in young mice. RTL1000 also inhibited splenic atrophy in both age groups. Additionally, there were major differences in splenocyte activation and cytokine secretion between younger and older mice in response to stroke. In conclusion, age-specific differences in the immune response to stroke resulted in RTL protection from experimental stroke through peripheral-based immune regulation in young mice and inflammatory tissue-specific protection of older mice.

### Conflict of interest statement

Dr. Offner, Dr. Alkayed and OHSU have a significant financial interest in Artielle Immunotherapeutics, Inc., a company that may have a commercial interest in the results of this research and technology. This potential conflict of interest has been reviewed and managed by the OHSU and VAMC Conflict of Interest in Research Committees. Dr. Offner discloses US patent #8,491,913 B2 for the use of recombinant molecules in treatment of stroke. The reviewer Dr. Dahan declares that, despite having collaborated with Dr. Offner, the review process was handled objectively.

## Abbreviations

RTL: recombinant T-cell receptor ligand
MCAO: middle cerebral artery occlusion
Treg: regulatory T cell
MHC: Major Histocompatibility Complex
HLA: Human leukocyte antigen
MOG: myelin oligodendrocyte glycoprotein
LDF: Laser Doppler flowmetry
PHA: Phytohemagglutinin
PMA: Phorbol myristate acetate.
